# Progesterone Interactions with the Cervix: Translational Implications for Term and Preterm Birth

**DOI:** 10.1155/2011/353297

**Published:** 2011-10-27

**Authors:** Bryan Larsen, Joseph Hwang

**Affiliations:** ^1^Marian University College of Osteopathic Medicine, Indianapolis, IN 46222-1997, USA; ^2^Iowa Perinatal Center, Mercy Medical Center, Des Moines, IA 50314, USA

## Abstract

The uterine cervix plays a vital role in maintaining pregnancy and an equally important role in allowing parturition to occur. Progesterone, either endogenously produced or supplied exogenously, supports the function of the cervix in sustaining intrauterine pregnancy, and the withdrawal of progesterone, either through natural processes or pharmacologic intervention, leads to delivery which underscores the importance of the progesterone's biological activities manifest in normal gestation and pregnancy that ends prematurely. Research crossing many scientific disciplines has demonstrated that progesterone is a pleotropic compound that affects the cervix through cytoplasmic and membrane receptors with profound effects on cellular and molecular functions that influence inflammatory cascades and extracellular matrix, both of which have consequences for parturition. Beyond the local cell and molecular biology of progesterone, it has systemic effects of relevance to pregnancy as well. This paper examines the biology of the cervix from its gross to cellular structure and biological activities of its cell and molecular processes that may be affected by progesterone. The implications of these processes for preterm birth are explored, and direction of current research is in relation to translational medicine implications for diagnostic, prognostic, and therapeutic approaches to threatened preterm birth.

## 1. Introduction

Yellon and coworkers [1] have aptly referred to the cervix as the “gatekeeper for pregnancy.” This does not minimize the role of uterine and placental biology, maternal-fetal interactions, and the integrity of the fetal membranes, nor does it abnegate neurobiological aspects of pregnancy maintenance [2]. The continuing concern for the persistently high rates of preterm birth that plague research-rich and research-poor countries [3] strategies that have a reasonable level of evidence for preventing untimely birth is a topic that deserves investigation. Indeed, as recent research has indicated that progesterone administered systemically [4] or locally [5] to forestall preterm birth (albeit with limitations and qualifications) has intensified the importance of understanding the relationship between progesterone and the cervix of the pregnant uterus.

The intentional focus on the biology of the cervix in this paper is supported by the recent review by Word et al. [6] who make the point that current research has confirmed that the remodeling of the cervix is a slowly developing process that occurs over a length of time and that the progressive changes in the cervix precede the uterine contractions by several weeks in normal pregnancy. 

The gatekeeper function of the cervix is simultaneously structural and biological, and the breadth of its functions and interactions with other anatomic structures and biologic or pharmacologic mediators should not be ignored. However, in this paper, we propose to review what is known about the role of the cervix as a mechanical structure and how progesterone influences this function. We will next consider the cellular and molecular actions that are inherent in maintaining pregnancy and in responding to changing hormonal conditions during gestation. Finally, the concept of inflammation as the final common pathway to parturition will be examined both from the standpoint of term and preterm pregnancy and options for altering the inflammatory processes that play a role in preterm birth.

## 2. Structural and Functional Attributes of the Cervix

At the gross level, the change of the cervix early in pregnancy from a dense, rigid tissue with a closed os to parturition with a softened consistency, shortened distance from os to membranes, and effacement at the time of labor, is well known and usually understood as a process that predominantly involves changes in collagen. Recent studies have elaborated that concept and suggest a process that is complex and nuanced. Many of the studies related to the precise processes involved in the pregnancy-related changes to the cervix have necessarily involved animal model studies, especially when experimental protocols have been involved. In considering the role of gonadal steroids in cervical biology and in particular the central role of progesterone in maintaining pregnancy, there is support for the concept that rodents provide a reasonable model for cell biology of the cervix, particularly as it applies to the extracellular matrix [1]. A recent report suggests that tissue-related changes in human subjects may be evaluated through fine needle biopsy of the cervix and that these biopsies were not accompanied by significant risk [7]. Based on this paper, it is likely that many of the observations derived from rodent studies may be verified or replicated in human research participants.

Another gross aspect of the cervical geometry through pregnancy is the shortening of the cervix. This has taken on a very important significance in recent years as the availability of transvagininal ultrasonography has been able to document the length of the cervical canal, and the length of the closed portion of the cervical canal relates to risk of preterm birth as summarized by recent reviews [8, 9]. Remodeling of the cervix and shortening of the cervical canal may be harbingers of precipitous birth both on the basis of loss of mechanical support, and also because the bacterial flora of the lower genital tract is moved closer to the fetal membranes overlaying the internal os.

As we will discuss later, the connection between amniotic infection and inflammatory reactions has been linked to preterm birth, and the role of shortened cervix is likewise associated. For example, Vaisbuch et al. correlated short cervix with amniotic inflammatory reaction, though not necessarily positive amniotic fluid culture, and 40% of those with intra-amniotic inflammation delivered within one week [10]. Even in the absence of documented infection, the inflammatory process might develop in response to bacterial exposure and possibly due to translocation of bacteria. Romero's group indicated that the development of sonographically identifiable collections of material referred to as “amniotic sludge” accumulated above the internal os and was associated with adverse pregnancy outcome [11]. Though the concept of sludge is relatively new, some of the developing literature on the topic suggests that sludge may contain bacteria as suggested by a report on the presence of sludge among short-cervix patients who also had intra-amniotic *Fusobacterium nucleatum* [12]. This favors the idea that the short cervix may enhance bacterial exposure. Of course, it did not take the invention of ultrasound for observant physicians to associate midtrimester ripening of the cervix with preterm birth risk and the idea of providing additional support for the mechanical attributes of the cervix in maintaining pregnancy. The idea of the cervix as mainly a mechanical gateway to parturition naturally leads to the concept of mechanical reinforcement of the cervix to overcome the functional deficiency in the structure. Cerclage has been used for decades to address this issue and has always been somewhat controversial, and it has fallen in and out of favor through the history of modern obstetrics. It is beyond the scope of this paper to review the current role of cerclage in obstetrical practice, but it does raise the related possibility that the cellular and molecular remodeling of the cervix through physiologic processes (term or preterm) may differ among individual patients. It is also possible that there may be a distinction between mechanical insufficiency of the cervix and premature labor which is mediated by irreversible biochemical changes allowing for the finding that indomethacin may stratify patients with short cervix [13].

Thus, cerclage is not uniformly effective in preventing delivery before 35 weeks, and with sufficient understanding of epidemiologic and biochemical markers involved in patients with sonographically documented short cervix, the application of cervical cerclage may be applied on a more individualized basis with improved outcomes. For example, cervical funneling may remain after cerclage, and one study found that when it does, the patient has a higher likelihood of delivering preterm than when funneling is not present [14]. 

Beyond the geometry, physical strength, pliability of the cervix, and the shape of the cervical canal during pregnancy, the cellular structure and molecular biology of the cervical cells will necessarily contribute to the mechanical attributes. It is recognized that progesterone has a demonstrable effect on the rate of cervical shortening and preterm delivery when administered to women admitted for preterm labor between 25 and 33 + 6 weeks gestation [15]. The cellular and molecular effects of progesterone on cervical biology will therefore be reviewed.

## 3. Cell Biology of the Cervix

Several cellular components of the cervix deserve mention. Importantly, the muscular character of the cervix is in many ways subordinate to the extracellular matrix in which connective tissue is a key to its mechanical strength. In a thorough review of the extracellular matrix of the body of the cervix, House et al. [16] emphasize that two aspects of the cervical changes during pregnancy (softening and ripening) should be individually understood as the former relates to the consistency of the cervical tissue which is progressively changing through pregnancy, and the latter relates to the load-bearing capacity of the cervix at the end of gestation. The ripening has been associated with the inflammatory activities which are commonly described as the final common pathway to parturition.

The significance of fibrillar collagen as the key component of the mechanical properties of the cervix is emphasized by its abundance and diminution in cervical insufficiency [17]. The body of the cervix includes fibroblasts, epithelial cells, and smooth muscle. The softening part of the remodeling process involves these cells over the course of pregnancy. In addition to collagen, other stromal components of the cervix include water which increases slightly throughout pregnancy, proteoglycans which appear to have a role in tissue hydration and association with collagen fibrils decreases during pregnancy, and elastin which may play a complementary role to collagen [16]. 

Many of the experimental studies on the composition and changes through the cervical remodeling process have been established in rodents in which gene knockout experiments [18], steroid hormone receptor blockade [19], or gene expression experiments [20] can be more readily and ethically accommodated than among humans. Thus, hyaluronan, which is a large anionic polysaccharide regulated by progesterone that has been studied extensively in rodents [21], is associated with proinflammatory activities interaction with collagen and glycopeptide versican. Timmons and Mahendroo [21] suggest that t he versican/hyaluron structures interpolated into collagen make the tissue more compliant that when collagen dominates. At parturition, hyaluronidase is increased in the cervix and cervical mucus, and the resulting breakdown of hyaluron allows for massive disaggregation of the biopolymers allowing for significant dilation/effacement of the cervix.

## 4. Progesterone and Cervical Function

Regulation of these elements of the body of the cervix involves, among other mediators, progestational agents. The presence and absence of progesterone and progestational agents are both critical at various stages of pregnancy. Maintaining pregnancy at the level of placenta, uterine quiescence, and limiting cervical inflammatory reactions are well-known attributes of progesterone. Progesterone withdrawal is a key element of parturition and is accompanied by a different set of biological cascades. As noted previously, attention is focused on progesterone because of recently concluded research on its potential use in preventing premature birth. Nevertheless, it is important in exploring the biological effects of progesterone to recognize its integration into systems that include multiple steroid and peptide hormones and tissues other than the cervix. 

As collagen plays an important role in the compliance and tensile strength of the cervix and progression toward a compliant and ultimately ripened cervix, the role of progesterone is of interest. The cervix begins softening through a process of continuously advancing collagen reorganization of the collagen fibrillar structure accompanied by decreasing levels of collagen accessory proteins, thrombospondin 2, and tenascin C as well as lysyl hydroxylase which is important in collagen crosslinking as demonstrated in mouse studies [22]. This report found that collagen content did not change during pregnancy, but its organization did. The practical importance of these elements are illustrated by the finding that thrombospondin 2 deficiency in mice results in untimely cervical ripening [23]. It is unclear whether progesterone has a direct regulatory effect on thrombospondin 2 expression in the cervix, but in myometrial cell culture, progesterone increased thrombospondin 1 expression, while estradiol decreased it [24].

Tenascins are extracellular matrix proteins that also contribute to the strength of tissues such as the cervix, and while not all details of its role in the cervix throughout pregnancy are known, in mouse models of endometrial stroma culture, it was found that tenascin C expression was positively controlled indirectly by progesterone, possibly through IL-1 alpha or prostaglandins [25]. Tenascin C decreases during pregnancy in mice consistent with the cervical softening process and is consistent with the collagen disorganization process.

Another aspect of collagen disorganization is related to enzymatic action by the matrix metalloproteinases (MMPs). The MMP family of enzymes, including collagenases, gelatinases, and elastases, have recognized significance in tissue remodeling events including intrauterine implantation, invasion by tumors, and vascular problems. The source of the MMPs is often inflammatory cells, but these enzymes may also be elaborated by other cell types. Studies of rat cervix during pregnancy found MMP2 to be present in the stroma but not epithelium in term pregnancy, but its role in cervical ripening was unclear as it was also present in nonpregnant rats [26], and MMP8 appeared after parturition and was associated with neutrophil infiltration. Fibroblast cultures made from partal women showed elevated (compared to nonpregnant women) MMP 1, 3 [27]. The authors attributed the fibroblast-derived MMPs to signaling from inflammatory cells present during labor. While there is information indicating the upregulation of MMP9 by estrogen and progesterone by the uterine stroma [18], no comprehensive inventory of MMPs and relationship to steroid hormone levels is available. Further complicating the picture is the fact that the net enzyme activity within tissues is the result of a balance between the enzyme and its inhibitors [28].

The role of hyaluronidase and hyaluronon, and versican and an MMP, ADAMTS1, was noted previously [21]. As indicated by the work of House et al. [18], progestational agents are mediators of hyaluronan expression. Hyaluronan in the cervical tissues results from a balance between synthesis (hyaluronan synthase 2) and degradation (hyaluronidase). Hyaluronan synthase activity is suppressed by progesterone according to mouse studies [29] while hyaluronidase activity remains unchanged. Another glycosaminoglycan, versican, is also regulated by hormonal control [30], and its disintegrin, ADAMTS1, which disrupts hyaluonan-versican complexes also is increased by progesterone [31].

Finally, it should be noted that attention has also been drawn to the peptide hormone relaxin which is highly conserved among various species and has been associated with increased compliance in the pubic symphysis of animals [32] as well as changes in extracellular matrix [33]. However, an attempt to establish its relationship to progesterone in pregnant rat studies indicated that progesterone apparently exerts control over the relaxin [34], and in a human cell line (JAR cells) medroxyprogesterone acetate upregulated relaxin expression [35].

A complex and as yet incomplete picture of how the extracelluar matrix of the cervix and its individual macromolecular components are regulated throughout pregnancy and parturition undoubtedly implies a very key role for progesterone. Some progesterone-regulated processes are the result of direct effects, while others may be mediated though inflammatory mediators or peptide hormones or enzymes from stromal or inflammatory cells. Despite the variety and complexity of coordinated events related to birth, it is clear that the physiologic processes involved in labor and delivery involve the functional withdrawal of progesterone. As reviewed by Mesiano et al. [36], this may be accomplished by a change in nuclear progesterone receptor expression, though details of the process are yet to be elucidated in humans and may as suggested by Mendelson [37] involve progesterone inactivity due to molecular interaction with transcription factors, changes in progesterone receptor co-regulator expression, metabolic modification of progesterone in stromal tissues, or expression of different isoforms of the receptor that leads to inhibition of progesterone activity. This paper emphasized the important fact that in addition to the traditional mechanism of progesterone action through nuclear receptors, much of the complexity of progesterone action results from nongenomic activities of progesterone both at the level of the cervix and in other tissues such as myometrium.

## 5. Premature Cervical Ripening

Cervical ripening is an element of preterm as well as term birth but raises the question as to the underlying mechanism of premature ripening and the corollary question of whether the mechanisms involved in premature ripening differ or are the same as those involved in term ripening of the cervix. Much of our current understanding has been gained through research completed in the past few years.

As mentioned before, the onset of labor has been associated with the presence of elevated cytokines, the influx of neutrophils, and activation of macrophages which has led to the often-repeated statement that inflammation appears to be the final common pathway for parturition. For example, cervical biopsies from uninfected term and preterm laboring women showed upregulation of Il-6, Il-8, and MCP-1 with elevated peripheral white counts and C-reactive protein which was interpreted as a common pathway in preterm as well as term labor [38]. Nevertheless, it is possible that there are nuances in the initial trigger for cervical ripening and mediators involved in the process at different gestational ages. The use of microarray analysis allowed the comparison of gene expression in mice at different gestational ages with premature delivery induced by the progesterone receptor inhibitor mifepristone, or by lipopolysaccharide [39]. Although the results indicated more than 300 genes were differentially regulated between experimental groups, the authors concluded that, indeed, there were distinct molecular mechanisms between term and preterm cervical ripening. A more focused evaluation of cervical biopsy-derived pro- and anti-inflammatory cytokine expression in women [40] who were in preterm or term labor, not in labor, or not pregnant, concluded that there were differences between preterm labor related to infection versus noninfected and overall differences between term and preterm cervical cytokine expression pointed to anti-inflammatory cytokines as key actors.

New information on the distinctive attributes of term and preterm cervical biology during parturition and differences in initiation of ripening process was published by Holt and coworkers in 2011 [41], emerged from a very comprehensive study of the mouse cervix wherein birth was elicited by lipopolysaccharide or mifepristone. The authors stated that their data indicate that preterm birth is not simply an accelerated form of physiologic cervical ripening. They also suggested that there were differences in inflammatory infiltrates with monocytes dominating progesterone receptor antagonism, while neutrophils dominated lipopolysaccharide-mediated premature ripening. Early induction of prostaglandin was related to Ptgs(Cox)2 with LPS and Ptgs(Cox)1 with mifepristone. While this report emphasized that many aspects of mouse cervical ripening are conserved in humans, there is further need to determine if these important findings hold for human cervical physiology.

There is general agreement that some cases of premature cervical ripening and hence preterm birth are related to infection and that infection may account for up to one-third of preterm births. The inflammatory response elicited by infectious agents has also become the accepted mechanism for preterm birth. In contrast, the remaining instances of preterm birth are attributed to less defined causes such as genetic background of the mother or fetus, environmental factors, intercurrent illness, or health conditions. In consideration of the focus on the cervix and the role of progesterone in maintaining and ending pregnancy, several recently discovered genetic associations that may have an impact on cervical ripening are reviewed.

Genetic associations with preterm birth imply, in some instances, potential effects on the cervix. Day and coworkers [42], reasoning that myometrial quiescence involves the activity of K+ channels and gene polymorphisms in mouse SK channel gene KCNN3 (small conductance calcium-activated potassium channel subfamily N, member 3) related to preterm and postdate birth, studied this gene in 602 families with at least 1 preterm birth. They associated six SNPs in this gene with preterm birth. While the role of this gene is mainly believed related to myometrial activity, the ion channels are involved in responses to stretch elicit Cox-2 activity and prostaglandin activity which affect the cervix as well as the myometrium. Thus, this genetic association may be predicted to have relevance to the cervix and, as the authors state, to other tissues as well. Anum and coworkers pursued an interesting approach to identifying candidate genes which may associate with preterm birth [43]. Reasoning that heritable diseases with genetic defects in connective tissue should result in preterm birth if these genes have a potential relationship, they were able to find a substantial number of genes that could contribute to cervical incompetence.

## 6. Cervical Epithelium

The cervical epithelium is composed of squamous cells on the external portion of the cervix and cuboidal cells within the os. The latter are more exposed during effacement. Because of the abundant microflora of the lower female genital tract the cervical epithelial cells will be exposed to indigenous bacteria throughout pregnancy and to exogenously acquired bacteria on specific occasions such as coitus during pregnancy. Because of the inflammatory events that are associated with preterm delivery, it is appropriate to consider whether the cervical epithelium is strictly a barrier to invasion by microorganisms, and whether the barrier function differs between squamous and columnar epithelium. Further, it is important to understand whether the cervical epithelia are able to sense and respond to exposure to microorganisms.

With regard to the barrier function of the epithelia, a recent report elaborates on the intercellular attachments of the squamous and cuboidal cells [44]. The endocervical columnar cells were found to have tight junctions that excluded fluorescently labeled IgG, whereas the superficial layers of the exctocervix lacked classical cell adhesion mechanisms and were permeable to labeled macromolecular probes, but below the apical-most layers, strong exclusionary junctions were present.

Despite the physical barrier, there are of course microorganisms that have the ability to invade through cells including a number of sexually transmitted pathogens, such as *Neisseria gonorrhea* and *Chlamydia trachomatis*, which will not be reviewed here. Of perhaps greater importance to the topic at hand is the possibility that the cervical epithelial cells can sense and respond to microbial molecules, and this response can elicit or at least contribute to initiation of the inflammatory cascade. Although immortalized tissue culture cells were used, Herbst-Kralovetz et al. demonstrated that endocervical and ectocervical epithelial cells possessed a full complement of toll-like receptors, and liganding these receptors with appropriate agonists could elicit a cytokine response [45], but some of the responses were not robust. An earlier study indicated that TLR4 was absent or perhaps deficient in endocervical cells, but CD14 coreceptor activity allowed these cells to respond to exposure to bacterial lipopolysaccharide [46]. 

The toll-like receptors are membrane-associated pattern recognition receptors (PRRs) that are foundational to innate immunity as they bind to commonly expressed microbial components (pathogenicity-associated molecular patterns or PAMPs) and transduce signals that elicit cytokine production. Less well studied are the NOD (nuclear oligomerization domain) receptors which are cytosolic receptors that bind bacterial cell wall components and also transduce signals that result in cytokine expression. The NODs are found in epithelial cells and would be expected to be part of the genital tract epithelium innate defense. Indeed, they have been found in functional form in endometrial epithelium [47] and in trophoblast cells [48] and would be anticipated to be present in endocervix, though this has not yet been confirmed.

Together, these reports confirm the ability of cervical epithelial cells to respond to microbial substances with cytokine reactivity. While it may make little difference whether cytokines generated in the lower female genital tract originate with the cervical stroma or epithelium, the host clearly has the ability to generate typical responses to microbial challenges with resulting cytokine release and subsequent cervical ripening and birth. But the question of how these receptors and responses are regulated remains. Progesterone was associated with decreased TLR-4 and TLR-9 signaling in macrophages [49, 50]. In a study of human tissues, TLR-2 and TLR-4 increased during pregnancy, and TLR-2 was especially elevated in labor [51], and treatment with medroxyprogesterone acetate decreased the ability of lipopolysaccharide to trigger a cytokine response. This effect may involve macrophages [50] but would not exclude cytokine downmodulation in other tissues, and the effect may be mediated rather than a direct effect on the TLR expression. Additional work is warranted to understand how these factors operate in preterm compared to term birth.

## 7. Cervical Inflammatory Reactions and Preterm Birth

Current medical literature repeatedly states that inflammation is the final common pathway to parturition. In considering what inflammatory activities are relevant to this concept, however, it is important to note that mechanistic details may differ with the gestational age at which preterm birth occurs, and there may be unique characteristics associated with inflammation that is centered in the placenta versus fetal membranes or the cervix. And current concepts emphasize that inflammatory reactions associated with preterm birth do not have to be focused in the generative tract tissues. For example, the inflammation associated with periodontitis during pregnancy has been linked to the risk of prematurity according to a recent review of more than 100 clinical studies [52]. The association of urinary tract infection and asymptomatic bacteriuria with low birthweight is well established [53], and even the fetus may participate in the inflammatory processes prior to birth [54] as illustrated by the fetal inflammatory response syndrome. Thus, while the focus of this paper is on the cervix and its responses to progesterone, the inflammatory reactions occurring at the time of parturition may exert effects in a variety of tissues. As an example, a study performed in pregnant mice treated intraperitoneally with low-dose lipopolysaccharide showed elevated cytokine levels in the maternal serum, but later, the cytokines appeared in the uterus, placenta, fetal membranes, and fetal serum [55]. 

While the literature on cytokine expression in relationship to preterm birth has grown substantially over the past decade, it is important to point out in relationship to the mechanism of preterm birth that the excursions in cytokine concentration are probably neither the first cause nor the final mechanism for delivery at the level of the cervix, uterus, or placenta. A recitation of the studies measuring cytokines in tissues and fluids at various gestational ages in term and preterm labor and delivery is not needed here. It will suffice to mention that a recent review [56] identified increased levels of Il-6, Il-8, Il-1*β*, and TNF*α* as associated with preterm birth, though the review also emphasized inconsistencies that remain in the literature. Thus, cytokines along with other biomolecules may most accurately be portrayed as indicators of the induction of the inflammatory cascade. 

If the cytokines serve as biomarkers, the natural question that follows is how they may support understanding and prediction of the clinical outcomes that may be imminent. One study indicated that IL-6 along with the other inflammatory marker C-reactive protein (CRP) levels predicted that a baby was more likely to be born with sepsis and that the time to delivery was related to these factors as well [57]. Elevated Il-8 obtained from amniotic fluid was a correlate of intra-amniotic microorganisms and histologic chorioamnionitis [58], and similarly, bacterial 16s ribosomal RNA was found in placentas of women who had elevated Il-6 and Il-8 [59]. While cord blood Il-8 was elevated in cases of funisitis and both Il-6 and Il-8 were present with histologic chorioamnionitis, the inflammatory marker MMP-8, a leukocyte collagenase, was not correlated [60]. Further detail about outcomes was reported recently when elevated maternal CRP and Il-6 but not MMP-9 were linked both with prematurity before 32 weeks and intraventricular hemorrhage in the infant.

While CRP and leukocytosis represent inflammatory markers linked to preterm birth in addition to interleukins, additional factors are also reported as being associated with preterm birth. Thus, macrophage inhibitory factor (MIP) has been associated with preterm birth [61] as were decreased levels of placental growth factor [62]. 

If early cytokine elevation serves as a biomarker for preterm birth, then cytokine origin deserves consideration. Cellular upregulation of cytokine expression is usually the result of cellular (surface or cytoplasmic receptor) components of the innate immune system that recognize pathogen-associated molecular patterns. These PRRs (pathogen recognition receptors) engage signaling cascades that result in cytokines that recruit leukocytes or activate cells of the immune system. Thus, microorganisms may be the initiating factor for proinflammatory cytokine production, but they are not the only source of cytokine generation. 

A cognate concept of PAMPs which are microbial-focused recognition systems is the DAMP or damage-associated molecular pattern that may play a role in recognition of endogenous molecules which have undergone alteration through processes such as necrotic damage or oxidation [63, 64]. The DAMP engages adapter molecules that can then activate signal transduction pathways. The role of specific adapter molecules RAGE (receptor for advanced glycation endproducts) and HMGB-1 (high mobility group box protein 1) has been examined in relationship to cervical ripening recently [65] and provides a potential clue about activation of inflammatory cascades in the absence of a microbial trigger.

Since proinflammatory cytokines do not represent the ultimate step in parturition, it is appropriate to note the downstream effects of these molecules. Increases in Il-6 and Il-8 at the cervix are associated with effacement [66]. In addition, TNF*α* and infiltrating leukocytes [67], are elevated in the ripening cervix, and ICAM (intercellular adhesion molecule) is overexpressed in vascular endothelium in preterm birth [68]. The cytokines expressed locally are involved in complex interactions with prostaglandin and nitric oxide which is considered to be a final mediator in the cervical maturation process [69]. 

Evidence has been presented that in the rodent uterus TLR-4 signaling after lipopolysaccharide binding induces MAP kinase signaling resulting in cyclooxygenase 2 (Cox 2) and intracellular phospholipase A(2) induction along with a number of other inflammatory proteins through Nf-*κ*B mediated transcription [70]. Interestingly Dubicke et al. found that rapid functional progesterone withdrawal by mifepristone treatment of mice upregulated Pgts1 (Cox 1) [40]. Also in pregnant mice, lipopolysaccharide induced prostaglandins (PGs) and nitric oxide (NO) in tandem and inhibitors diminished both PG and NO as well as birth [71]. But the same report also indicated that NO increased or decreased PG levels according to the NO concentration in direct relationship. Despite the similar kinetics of NO and PG, they each may have distinct activities in the uterus versus cervix and may be differently regulated [69, 72]. 

The majority of the discussion related to cervical inflammatory reactions and term and preterm birth have centered on the proinflammatory cytokines with emphasis primarily focusing on IL-1*α* TNF*α*, Il-6, and Il-8, and the modulation of cytokine-mediated biological activity may occur through the anti-inflammatory cytokines. The current literature has tended to elevate Il-10 as the most significant anti-inflammatory cytokine in relation to preterm birth to date. In mouse pregnancy, preterm birth tended to be characterized by increased levels of several proinflammatory factors, while there was a downregulation of Il-10 [73]. Likewise in chorionic villous cultures from early miscarriages, Il-10 was attenuated while IL-1a and Il-6 were elevated [74]. In the presence of intra-amniotic infection at term, there was a higher level of amniotic fluid Il-10 than among term pregnancies without infection, and in both term and preterm birth, the Il-10 was elevated [75] which on the surface seems counterintuitive but may reflect a feedback mechanism to dampen the proinflammatory reaction after parturition. A large-scale evaluation of cord blood cytokines in preterm and term birth showed elevations Il-10 at the same time Il-2, 4, 5, and 8 along with MCP-1, MIP1*α* and *β*, soluble Il-6 receptor a, TNF*α*, soluble TNF*α* and TREM-1 which were also elevated [76].

Another approach to examining inflammatory modulation has been to experimentally use substances that antagonize pro-inflammatory cytokines. A study involving 75 women with recurrent spontaneous abortion showed improved live birth rates when etanercept or adlimumab were added to a regimen of anticoagulant and IVIG [77]. Though not having direct clinical application, a study of placental explants challenged with bacterial components (lipolysaccharide or lipoteichoic acid) showed elevation of Il-6 and Il-8 with a decrease in most inflammatory cytokines and Prostaglandin E(2) when Il-10 was added to the incubation medium (78 19821803).

Recognizing that there are other anti-inflammatory factors, some investigators have established balance sheets that attempt to determine if broad panels of cytokines favor pro- or anti-inflammatory cytokines. One report indicated a 7-fold increase in preterm birth (before 34 weeks) risk when a high inflammatory to anti-inflammatory ration was detected [78]. Other studies agree, indicating that most pro-inflammatory cytokines are increased in preterm birth, while anti-inflammatory cytokines are decreased [79], and specifically in the cervix the pro-inflammatory versus anti-inflammatory score predominated [80].

Before leaving the topic of the interplay between pro- and anti-inflammatory cytokines, brief mention should be made regarding the genetic background of individuals that could contribute to adverse pregnancy outcomes. Gene polymorphisms have been identified in structural genes of inflammatory factors or their receptors which can lead to defective bioactive molecules or even complete absence of the gene product. In addition, many single-nucleotide polymorphisms located in gene promoter regions of inflammatory cytokines have been identified which can alter the regulation and hence the response to normal stimuli [81, 82]. In the present context, the finding that several polymorphisms in the gene for Il-10 receptor were associated with the concentration of IL-10 in the cervix suggests that both feedback and feed-forward arms of the inflammatory cascade will probably be important for cytokine expression, but crosstalk between cytokines and other biologically active compounds. A mutation in fetal Il-10 (-1082A) was associated with infection-related preterm birth [83] as were other gene polymorphisms in mother and fetus. As we have seen in the past few years, the complexity of these regulatory networks is continuing to be elucidated, and additional nuances of these networks are being discovered.

## 8. Progesterone and the Inflammatory Cascade

At the outset of this paper, two concepts were mentioned. First, inflammation is involved as a common pathway in birth, and second, progesterone (endogenous or exogenous) is one of the most consistently effective compounds in maintaining pregnancy, namely, averting the initiation of the common pathway of labor. The two concepts now come together in the following paragraphs.

 Progesterone has often been described as having immune-suppressive activity and from a teleological point of view, this makes sense as the mother's immune system needs to be tilted toward tolerance to host the fetal allograft. Recent reviews have noted that the presence of sex steroid receptors in innate immune cells such as macrophages or lymphocytes, is well recognized [84], but innate immune responses also involve epithelial and stromal cells such as those found in the cervix. Smooth muscle was able to act both as contractile cells and innate immune cell as stimulation of smooth muscle cells (uterine and cervical) with IL-1*β* showed through microarray-based analysis that 38 inflammation and immunity genes were stimulated [85]. Sex steroids affect both immune and epithelial cells and are able to elicit various growth factors from stromal cells [86].

Progesterone does not act in isolation from other steroid and peptide hormones, and its activities are made more pleotropic as a result of the fact that it has biological activity resulting from traditional gene level activation through steroid response elements on steroid hormone-regulated genes, but also nongenomic activities mediated by membrane receptors which engage in G-coupled signaling cascades. Many of the interactions with the innate immune system, and by extension, term and preterm parturition appear dependent on this non-genomic mode of cellular activation. 

Adding to the complexity and hence the lack of absolute clarity in the precise mechanism of progesterone action on the immune system is the fact that some of the activities of progesterone are systemic and some are local. For example, a study of 17*β*-OH steroid dehydrogenase which converts estradiol to the less estrogenic estrone and 20*α*-OH progesterone to the more active progesterone showed that at parturition, this enzyme was downregulated in endocervical cells allowing for a localized functional progesterone withdrawal [87]. Studies of isolated cells have the benefit of examining the effects of compounds added to cell cultures on the biology of those cells but have the simultaneous disadvantage of not showing what happens when those same cells are integrated into multicellular organ systems. MAP Kinase signaling cascades have been demonstrated as characteristic for the cervical ripening process [88] in humans. The introduction of 17*α*-hydroxyprogesterone caproate treatment randomly assigned to pregnant women decreased cervical shortening after 21 days and decreased IL-1b but did not affect Il-6, Il-8, TNF*α*, or nitric oxide [89]. These studies raise the question of whether progesterone affects the trafficking or maturation of inflammatory cells which produce inflammatory cytokines and chemokines, or whether the effects occur more immediately on the cell level processes that elicit the inflammatory mediators. Given the pleotropic nature of the progesterone, it is likely that a long list of direct and mediated effects can be described.

In a pregnant rat study [90], normal pregnancy proceeded with decrease in inducible nitric oxide synthase and an increase in COX-2. But with progesterone inhibition (onapristone), expression of both enzymes increased as premature birth was initiated. There is reason to believe that these effects could at least be partly explained by progesterone effects on inflammatory cells. Dubicke et al. and coworkers [40] characterized the cellular infiltrates during parturition induced by progesterone withdrawal (mifepristone) or lipopolysaccharide and showed that the latter induced a neutrophil response and the former favored a monocytic response. Thus, the effects of progesterone may also depend on the mechanism initiating inflammatory reactions. In another study involving 17*α*-hydroxyprogesterone caproate, treatment did not affect basal levels, but with stimulation by lipopolysaccharide or lipoteichoic acid, Il-6 was elevated, whereas progesterone-treated subjects did not have elevated cytokine [91].

The possibility that some of the effects of progesterone are initiated by effects on inflammatory cells is suggested by the finding that progesterone can modify TLR signaling. Macrophages treated with progesterone had diminished responses to stimuli that act through TLR4 and TLR9 [49, 50] with decreased IL-6, and iNOS, and TLR4 expression. In lipopolysaccharide-stimulated macrophages, progesterone treatment increased expression of SOCS-1 (suppressor of cytokine signaling-1), suggesting a possible mechanism for the progesterone effect. This is in consonance with an earlier observation that progesterone decreases mouse macrophage nitric oxide [92].

Progesterone is responsible for interactions with leukocytes which elaborate progesterone-induced blocking factor [93] which has immunomodulatory functions. Activated pregnancy lymphocytes have membrane receptors for progesterone, and the action of PIBF under control of progesterone increases Th2 type signaling by a novel Il-4 receptor that in mouse studies activated the JAK/STAT signaling pathway [94] accompanied by a decrease in phospholipase A2 which decreases prostaglandin production. Although studied extensively in mice, PIBF is also produced in human pregnancy, and its effects are consistent with beneficial effects on continuing gestation. 

The possibility that progesterone exerts effects on the immune network at a central regulatory level is suggested by studies that indicated T-regulatory cells (CD4^+^ CD25^+^) are increased peripherally and in the uterus of progesterone-treated mice [95] and by a study in humans that found T-regulatory cells were upregulated in the midtrimester. These regulatory cells suppressed Il-2, TNF*α*, and gamma interferon, while upregulating Il-4 and Il-10 in responder cells.

Progesterone effects on the immune network have been noted in a number of studies going back many years. Progesterone has been associated with inhibitory effects on T cells, macrophages, and natural killer cells [96]. Normal, uncomplicated pregnancy shows a decrease in proinflammatory cytokines as pregnancy progresses, and increasing anti-inflammatory factors is indicative of immune regulation in the physiologic adaptation to gestation [97]. Th2 cytokines are favored by normal pregnancy [98], and a study a herbal traditional medicine treatment for threatened abortion showed a shift from Th1 to Th2 responses with a correlation with Il-10 [99]. 

Keeping the focus of this paper on the role of the cervix and its interaction with progesterone, it is impossible to ignore the important activities it has in maintaining uterine quiescence and effects on myometrial activity, or the interaction with the inflammatory activity at the decidual interface between mother and fetus. Effects on vascular endothelium, also mediated through inflammatory factors relative to uterine blood flow, have been a topic of interest for researchers and in some instances help inform the possible actions in the cervix during pregnancy. Indeed, many of the non-genomic effects of progesterone have been discovered through studies of the uterus rather than the cervix. Many of the non-genomic effects of progesterone (and estrogen) have been focused on the potential role of hormones in cancer and metastatic processes. These various other attributes of progesterone have proved important in recognizing that cell surface receptors for progesterone can transduce signals through the MAP kinase pathway including vascular functions [100, 101]. Evidence of progesterone signaling was reported in breast cancer research [102]. More directly related to the focus on the cervix, MAP kinase signaling by progesterone was able to affect the expression of extracellular matrix-related integrin synthesis which clearly has implications for the cervical ripening process [103]. The ultimate expression of factors induced by signal transduction will involve nuclear transcription factors, and in this regard, it is notable that progesterone was found to suppress the NF-*κ*B expression resulting in depressed cytokine levels [104].

The complexities of progesterone activities biological mechanisms related to pregnancy and parturition have not been exhaustively reviewed here, but it is clear that there are several mechanisms known to be consistent with the forestalling of parturition. However, additional details continue to be uncovered. For example, progesterone affects monocytes at the earliest level of pregnancy through means of glycodelin which has been recently reviewed and has activities that may contribute to the Th2 dominance in pregnancy [105]. While glycodelin A was studied in relation to implantation, its effect on MMP2 and MMP9 may be of importance to the prevention of untimely cervical ripening. Glycodelin A has been identified in 11 of 14 archival specimens of cervical epithelium [106], but its role in the pregnant cervix is only conjectural at present. Thus, the understanding of the precise role of progesterone in support of pregnancy and the details as expressed in various tissues will only continue to become more complex as its multiplicities of function are investigated.

## 9. Translational Implications

A substantial body of scientific knowledge has been built around the concept that the uterine cervix serves a physical support function for the gravid uterus changing from a rigid to a pliable structure at the time of delivery. But in the absence of information that brings some practical value to the extensive research reported here, the understanding of the physiology, cellular biology, and environmental effects on cervical function will be strictly academic.

The structural role of the cervix, like other structures, is only part of the story. The softening and dilation of the cervix is a biological process timed intricately, coinciding with the uterine contractions that move the fetus into and through the birth canal. This can, and too often does, occur prematurely. Sometimes, the biological characteristics recede into the background as the structural properties of the cervix assume the central focus. In the case of multiple births, cervical dysfunction seems largely a matter of structural support. The same is also true when cervical dysfunction is secondary to previous LEEP procedure. Despite the conceptual potential value of cerclage in support of the structural function, the role of cerclage has not proved uniformly successful, and in evidence-based medicine studies, its use has limited endorsement [107]. 

The fact that cerclage is not uniformly successful in preventing premature cervical ripening and delivery attests to the fact that biological processes are also at work. The process of parturition engages many of the mediators and consequences of inflammation, and these have profound effects on the uterus and the cervix. We emphasized that within the cervix the inflammatory cascade is highly active during cervical ripening and involves inflammatory cells and mediators which lead to remodeling of the tissue. The process leads to increased tissue compliance through alterations in extracellular matrix coupled with proteolytic action in the tissue and finally with the production of prostaglandins and nitric oxide which are the final steps in a cascade process. Since we recognize the final pathway to birth as a cascade process, it may not matter what specific event triggered the final stages of cervical ripening, labor, and delivery as the process is likely to proceed despite tocolytic drugs although delivery might be delayed slightly to allow for antenatal steroid use. 

The ability to determine the presence of a shortening of the cervical canal has created a new means of predicting the likelihood of engaging the inflammatory process prematurely. This finding of a physical change in the geometry of the cervix can be related to the mechanism of prematurely engaging the inflammatory process at least theoretically because of the partial disruption of cervical mucus and the movement of the bacterial flora into closer proximity to the endocervical epithelium and its unique display of bacterial recognition receptors and to the fetal membranes where infection and inflammatory processes may arise. 

The significance of the shortened cervix has its translational application in the duality represented by structural compromise coupled with physiologic changes. Therefore, clinicians have considered both structural and pharmacologic approaches to the short cervix. Cerclage addresses the advancing compliance of the tissue, and progesterone has been introduced because of its demonstrated utility at least in a percentage of cases of impending preterm labor. The most recent compilation of the evidence for use of progesterone [108] concludes that progesterone helps prevent birth before 32 weeks and has benefits for newborn mortality and respiratory distress syndrome and necrotizing enterocolitis.

We have attempted to document the multiple levels at which progesterone affects the cervical biology in a variety of ways that promote the prolongation of pregnancy. Unfortunately, the scientific explanation of all of the effects of progesterone at the level of the cervix has not been established, but sufficient understanding is available to appreciate how this intervention may reduce the likelihood of adverse outcomes, although as many structured reviews have stated, the literature does not yet contain studies that adequately address all the practice questions related to the use of progesterone including adverse metabolic effects on mother and fetus. 

 Microorganisms may be the genesis of inflammatory reactions leading to preterm birth, and several investigators in large and small studies have sought the translational application of this fact in the hypothesis that antibiotics should prevent prematurity. However, evidence reviewed in 2007 did not show that antibiotic treatment of at risk women prevented preterm birth [109]. A different paper [110] concluded that macrolide or clindamycin given in the second trimester to women at risk for preterm birth did lower their risk of delivering prematurely but metronidazole increased risk. Once membranes were ruptured prematurely, the introduction of antibiotic treatment while not preventing premature birth provided several benefits including greater likelihood of delivering later than 48 hours after membrane rupture, and decreased chorioamnionitis [111]. The science suggesting that preterm birth is part of a cascade suggests that antibiotics are unlikely to alter the course of the inflammatory cascade once it has been initiated. 

Another lesson implied by the concept of inflammation-related cascades as the trigger for initiation of parturition is that we need to develop a better understanding of how to manage the trigger. Antibiotics in general terms represents a blunt instrument, and simply introducing antibiotics broadly into obstetric care cannot be expected to be effective as the literature has demonstrated. Every pregnant woman carries an abundant flora, and the flora differs among individuals. Some of the organisms found commonly in the flora have a reputation as harmful, and yet eliminating these organisms has not had an effect on prematurity [112]. Individuals will also have unique genetic backgrounds that are only beginning to be related to their immune responses to different microorganisms. We are still in the process of discovering microorganisms present in the normal flora that cannot be cultivated, but only detected through molecular methods. In addition, we still struggle with the urge to identify every organism as a pathogen or nonpathogen, which is an artificial and sometimes counterproductive way of looking at microorganisms. Based on better methods for unraveling the complexity of host-microbe interactions, new molecular targets will be developed in the future.

In this paper, we have also noted that recent research suggests that there are differences in some of the mechanistic details between birth that is related to progesterone withdrawal versus birth precipitated by bacterial triggering of virulence pattern recognition receptors such as TLR or NOD molecules. Continued research may identify useful targets in these pathways, but interestingly, information suggesting that progesterone may modulate the terminal steps in the delivery cascade (prostaglandin and nitric oxide) suggests that the role of progesterone may increase in the future, depending on the outcome of well-designed and appropriately powered studies to investigate efficacy, closely defined populations for study, and investigation of adverse effects in mothers and their infants after administration of pharmacologic interventions. Translational research will continue as an outgrowth of the science that identifies novel targets for interdicting preterm birth, but devising safe and meaningful research that provides useful evidence that truly informs clinical practice will be a challenge as investigators, clinicians, and funding agencies wrestle with deciding which approaches are most appropriate and promising for clinical trials.
